# COVID-19 Admission Rates and Changes in US Hospital Inpatient and Intensive Care Unit Occupancy

**DOI:** 10.1001/jamahealthforum.2023.4206

**Published:** 2023-12-01

**Authors:** Giacomo Meille, Sandra L. Decker, Pamela L. Owens, Thomas M. Selden

**Affiliations:** 1 Agency for Healthcare Research and Quality, US Department of Health and Human Services, Rockville, Maryland

## Abstract

**Importance:**

The COVID-19 pandemic had unprecedented effects on hospital occupancy, with consequences for hospital operations and patient care. Previous studies of occupancy during COVID-19 have been limited to small samples of hospitals.

**Objective:**

To measure the association between COVID-19 admission rates and hospital occupancy in different US areas and at different time periods during 2020.

**Design, Setting, and Participants:**

This cross-sectional study used data from the Healthcare Cost and Utilization Project State Inpatient Databases (2019-2020) for patients in nonfederal acute care hospitals in 45 US states, including the District of Columbia. Data analysis was performed between September 1, 2022, and April 30, 2023.

**Exposures:**

Each hospital and week in 2020 was categorized based on the number of COVID-19 admissions per 100 beds (<1 [low], 1-4.9, 5-9.9, 10-14.9, or ≥15 [high]).

**Main Outcomes and Measures:**

The main outcomes were inpatient and intensive care unit (ICU) occupancy. We used regression analysis to estimate the average change in occupancy for each hospital-week in 2020 relative to the same hospital week in 2019.

**Results:**

This study included 3960 hospitals and 54 355 916 admissions. Of the admissions in the 40 states used for race and ethnicity analyses, 15.7% were for Black patients, 12.9% were for Hispanic patients, 62.5% were for White patients, and 7.2% were for patients of other race or ethnicity; 1.7% of patients were missing these data. Weekly COVID-19 admission rates in 2020 were less than 4 per 100 beds for 63.9% of hospital-weeks and at least 10 in only 15.9% of hospital-weeks. Inpatient occupancy decreased by 12.7% (95% CI, 12.1% to 13.4%) during weeks with low COVID-19 admission rates and increased by 7.9% (95% CI, 6.8% to 9.0%) during weeks with high COVID-19 admission rates. Intensive care unit occupancy rates increased by 67.8% (95% CI, 60.5% to 75.3%) during weeks with high COVID-19 admissions. Increases in ICU occupancy were greatest when weighted to reflect the experience of Hispanic patients. Changes in occupancy were most pronounced early in the pandemic. During weeks with high COVID-19 admissions, occupancy decreased for many service lines, with occupancy by surgical patients declining by 43.1% (95% CI, 38.6% to 47.2%) early in the pandemic.

**Conclusions and Relevance:**

In this cross-sectional study of US hospital discharges in 45 states in 2020, hospital occupancy decreased during weeks with low COVID-19 admissions and increased during weeks with high COVID-19 admissions, with the largest changes occurring early in the pandemic. These findings suggest that surges in COVID-19 strained ICUs and were associated with large decreases in the number of surgical patients. These occupancy fluctuations may have affected quality of care and hospital finances.

## Key Points

**Question:**

Were COVID-19 admission rates associated with inpatient and intensive care unit (ICU) occupancy rates in 2020?

**Findings:**

In this cross-sectional study of 3960 US hospitals in 45 US states, weekly rates of COVID-19 admissions were less than 4 per 100 beds for 64% of hospital-weeks and at least 10 per 100 beds in 16% of hospital-weeks. During weeks with low COVID-19 admissions (<1), inpatient occupancy decreased by 13%; during weeks with high COVID-19 admissions (≥15), inpatient occupancy increased by 8% and ICU occupancy increased by 68%.

**Meaning:**

COVID-19 admission rates were associated with substantial changes in occupancy, with ICUs especially strained during surges.

## Teaser

This cross-sectional study examines COVID-19 hospital admissions and changes in inpatient and intensive care unit (ICU) occupancy for different US states and time periods during 2020.

## Introduction

There were large fluctuations in hospital occupancy during the COVID-19 pandemic. Factors such as deferrals of care decreased occupancy at some hospitals, whereas high COVID-19 caseloads strained others. These fluctuations may have affected health care workers,^1,2,3,4^ quality of care,^5,6,7,8,9,10,11,12^ and hospital financial stability.^13,14,15,16,17^ Understanding the burden of COVID-19 on hospitals may inform policy responses to future public health emergencies (PHEs).

Previous studies of US hospital occupancy during the COVID-19 pandemic have been limited to convenience samples of fewer than 300 hospitals and have not examined the association of occupancy with COVID-19 surges.^5,18,19,20,21^ To our knowledge, this study is the first analysis of hospital exposure to COVID-19 and its association with occupancy using hospitalizations from all nonfederal acute care hospitals in 45 states, including the District of Columbia. We measured changes in inpatient and intensive care unit (ICU) occupancy during periods of high and low COVID-19 admissions by comparing each hospital-week during the first 38 weeks of the PHE in 2020 to the same hospital-week in 2019. In addition to presenting national average estimates, we examined changes in occupancy by time period (early and late 2020), admission type, urbanicity, state, and patient race and ethnicity.

## Methods

### Data and Sample

This cross-sectional study used data from the Agency for Healthcare Research and Quality (AHRQ) Healthcare Cost and Utilization Project (HCUP) State Inpatient Databases (SIDs; 2019-2020) for 45 states, representing approximately 97% of US hospitalizations in nonfederal acute care hospitals. Alabama and Idaho did not participate in HCUP, and Colorado, Connecticut, Pennsylvania, and Washington were excluded because they did not provide admission dates. The data covered all patients (regardless of insurance coverage) discharged from all nonfederal acute care hospitals in the included states. Two additional variables, hospital urban or rural location and number of total and ICU beds, were merged from the American Hospital Association Annual Survey Database. Analyses related to patient race and ethnicity excluded 5 states that had more than 5% of these data missing (Louisiana, Montana, North Dakota, Nebraska, and West Virginia). We included all patients aged 1 year or older.

The AHRQ human protection administrator determined that this study did not constitute human participant research and therefore did not require institutional review board approval or informed consent. This study followed the Strengthening the Reporting of Observational Studies in Epidemiology (STROBE) reporting guideline.

### Primary Outcomes

Our outcomes were inpatient occupancy, ICU occupancy, and occupancy by service line at the hospital-week level (weeks were defined as consecutive 7-day periods beginning on January 1 and mapping February 29, 2020 to February 28, 2020). Occupancy rates were calculated using patient admission and discharge dates and dividing by the number of staffed beds in 2019. For the 33 states that reported revenue codes (eTable 1 in Supplement 1), we examined ICU occupancy using the number of ICU beds in 2019 (or, if missing, the maximum ICU occupancy in 2019) and identifying ICU stays with revenue center codes 0200 (general ICU), 0201 (surgical ICU), or 0202 (medical ICU). We used Major Diagnostic Categories (MDCs), Medicare Severity-Diagnostic Related Groups (MS-DRGs), and the HCUP Clinical Classification System Refined (CCSR) for *International Classification of Diseases, 10th Revision, Clinical Modification* (*ICD-10-CM*) diagnoses^22^ to assign each hospitalization to 1 of 5 hierarchical service lines: maternal (MDCs 14 and 15), mental health and substance use disorders (MDCs 19 and 20), injury (any CCSR in INJ001-INJ027 or INJ032), surgery (surgical-related MS-DRGs), and medical (all other hospitalizations).

### COVID-19 Admission Rate

Beginning on April 1, 2020, admissions were considered COVID-19 positive if they included *ICD-10-CM* code U07.1 or J12.82.^23^ Before April 1, 2020, admissions were considered COVID-19 positive if they included *ICD-10-CM* coronavirus code B97.29 and any of the following *ICD-10-CM* respiratory codes: J06.9, J12.89, J20.8, J21.89, J22, J40, J80, J98.8, R05, R06.02, or R50.9.^24^ The COVID-19 admission rate per 100 beds for each hospital-week was categorized using 5 groups (<1 [low], 1-4.9, 5-9.9, 10-14.9, or ≥15 [high]).

### Other Independent Measures

Additional independent variables included hospital urban or rural location, patient race and ethnicity, and time period. The HCUP databases rely on patient race and ethnicity as recorded on the discharge record by the hospital, which is ideally based on self-report but may be collected based on observation.^25,26^ Hospital location was categorized as large central metropolitan, other metropolitan, micropolitan, or rural.^27^ Patient race and ethnicity was categorized as Hispanic, non-Hispanic Black (hereinafter Black), non-Hispanic White (hereinafter White), or other non-Hispanic race or ethnicity (American Indian or Alaska Native, Asian or Pacific Islander, or multiple races or ethnicities). We divided 2020 into the pre-PHE period (weeks 1-10) and the PHE period (weeks 11-48). The PHE was the period of interest, as there were relatively few COVID-19 cases before the PHE was declared on March 13, 2020 (week 11). Data from the last 4 weeks of 2020 were excluded because patients discharged in 2021 were not included in the 2020 SIDs. In heterogeneity analyses, we split the PHE period into the early pandemic (weeks 11-26) and the second half of 2020 (weeks 27-48) because of policy differences, including lockdowns and recommendations to defer nonelective surgeries, as well as evolving testing availability and knowledge about treating COVID-19.

### Statistical Analysis

The association between rates of new COVID-19 admissions and hospital occupancy was measured by comparing each hospital and week in 2020 to the corresponding hospital-week in 2019. Using linear regression models and hospital-week fixed effects, occupancy rates were regressed on the categorical COVID-19 admission rate interacted with indicators for the pre-PHE and PHE periods. The categorical COVID-19 admission rate allowed for a nonlinear relationship between COVID-19 admission rates and occupancy rates. Standard errors were clustered at the hospital level. Most regressions weighted each hospital’s observations by 2019 total admissions. In some analyses, observations were weighted by 2019 hospital admissions for each racial or ethnic group, representing the average experience for patients who were Black, Hispanic, White, or of other race or ethnicity. Our findings should be interpreted as exploratory due to the potential for type I error and multiple comparisons.

Statistical significance was assessed at the *P* < .05 level using 2-tailed tests. All analyses were performed using Stata-MP, version 17 (StataCorp LLC) from September 1, 2022, to April 30, 2023.

## Results

### COVID-19 Admission Rates Across Hospitals and States

Inpatient occupancy was examined for 3960 hospitals in 45 US states (54 355 916 admissions in 393 580 hospital-weeks), and ICU occupancy was examined for 2703 hospitals in 33 states (34 170 634 admissions in 268 416 hospital-weeks). There were 751 hospitals (19.0%) in large metropolitan counties, 1624 (41.0%) in other metropolitan counties, 641 (16.2%) in micropolitan counties, and 944 (23.8%) in rural counties. Of the admissions in the 40 states used for race and ethnicity analyses, 15.7% were for Black patients, 12.9% were for Hispanic patients, 62.5% were for White patients, and 7.2% were for patients of other race or ethnicity; 1.7% of patients were missing these data.

**Figure 1.**
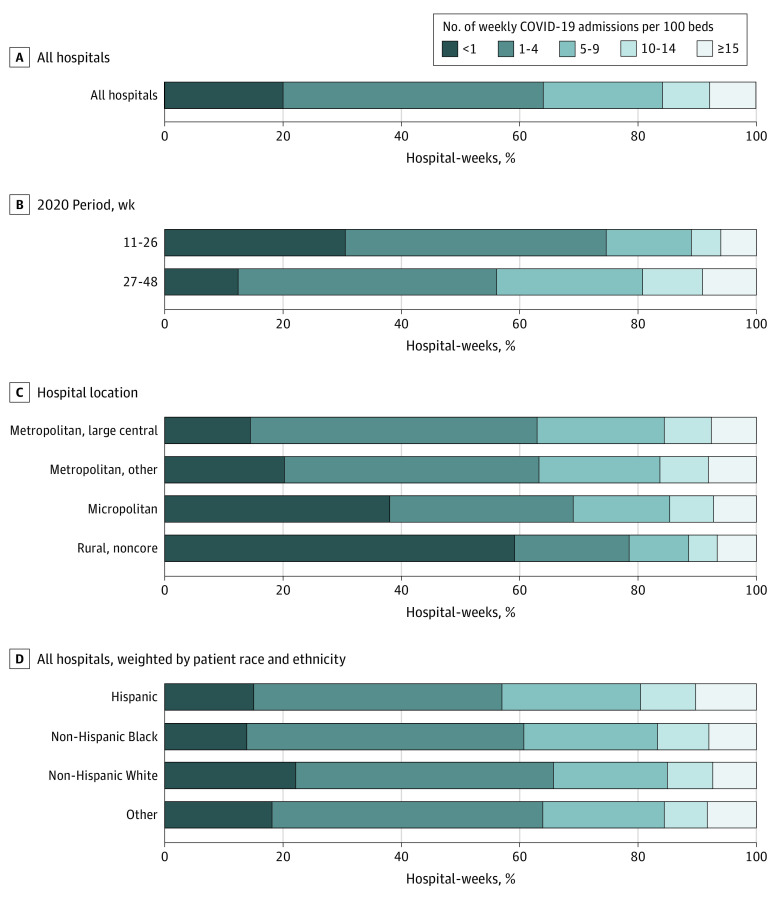
Distribution of Hospital-Weeks by COVID-19 Admission Rate, 2020 Weeks 11 to 48 A, All hospitals. B, Time period in 2020. C, Hospital location. D, Weighted by number of admissions in 2019 by patient race and ethnicity. Other race and ethnicity includes American Indian or Alaska Native, Asian or Pacific Islander, or multiple races or ethnicities. Analyses related to patient race and ethnicity excluded 5 states that had more than 5% of these data missing (Louisiana, Montana, North Dakota, Nebraska, and West Virginia). In A to D, all statistics were calculated using hospital-level weights equal to the number of admissions in 2019. For each panel, the reference category is the first one listed. For all other categories, the difference between their distribution and that of the reference category is statistically significant at the 1% level. Data were obtained from the Agency for Healthcare Research and Quality Healthcare Cost and Utilization Project State Inpatient Databases (2019-2020) for 45 US states.

Figure 1 presents the distribution of hospital-weeks by COVID-19 admission rate (full results in eTable 2 in Supplement 1). Periods with low (<1) or relatively low (1-4) COVID-19 admission rates accounted for nearly two-thirds of hospital-weeks in 2020, whereas periods with relatively high (10-14) or high (≥15) COVID-19 admission rates accounted for less than one-fifth of hospital-weeks. eFigure 1 in Supplement 1 shows that most periods of high COVID-19 admissions lasted less than a month, with 34.3% lasting 1 week, 49.8% lasting no more than 2 weeks, and 63.1% lasting no more than 3 weeks. Early in the pandemic, periods with low COVID-19 admissions were relatively common (30.5% of hospital-weeks), whereas periods with high COVID-19 admissions were infrequent (6.1%). During the second half of 2020, a smaller proportion of hospital-weeks had low COVID-19 admissions (12.3%), and a higher proportion had high COVID-19 admissions (9.2%). The proportion of hospitals with high COVID-19 admissions varied substantially by week, following a wave pattern and reaching as high as 33.9% at the end of 2020 (eFigure 2 in Supplement 1).

The proportion of hospital-weeks with low COVID-19 admissions was greater for rural hospitals (59.2% of hospital-weeks) compared with large metropolitan hospitals (14.5%). The proportion of hospital-weeks with high COVID-19 admissions was only slightly higher in large metropolitan areas compared with rural counties (7.7% vs 6.7%).

The final set of distributions in Figure 1 weighted all hospitals by the number of admissions for each race and ethnicity in 2019. On average, Black and Hispanic patients were less likely to be admitted to hospitals with low COVID-19 burden (13.9% and 15.1% of hospital-weeks, respectively) compared with White patients (22.2%). Black and Hispanic patients were slightly more likely to be admitted to hospitals with high COVID-19 admissions (8.1% and 10.4%, respectively) compared with White patients (7.5%).

**Figure 2.**
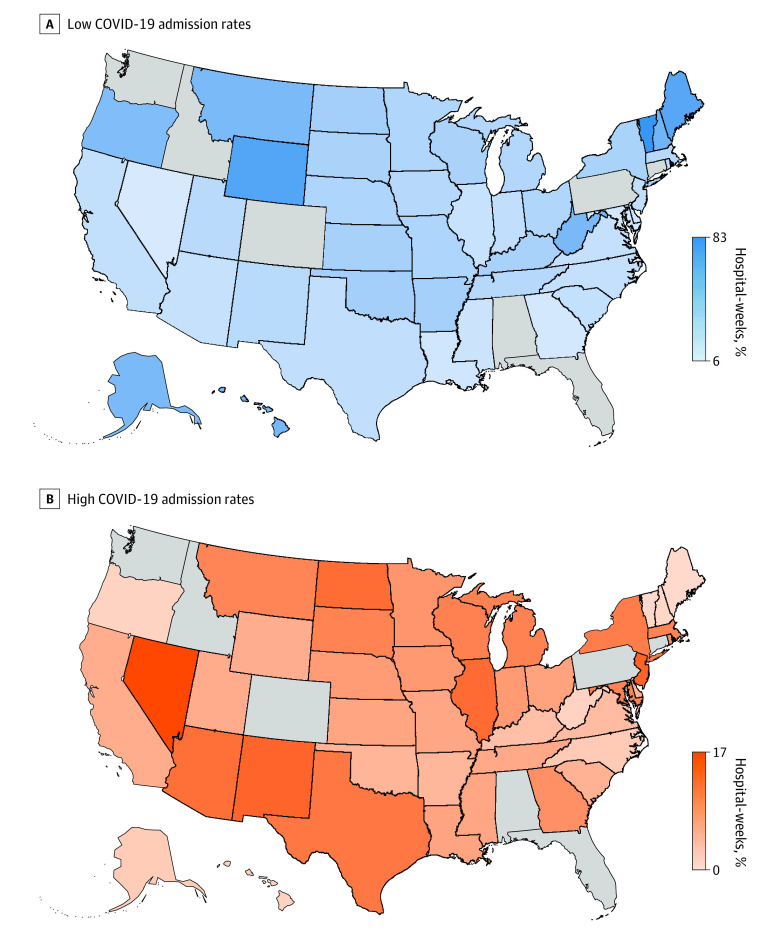
Hospital-Weeks With Low or High COVID-19 Admission Rates, 2020 Weeks 11 to 48 A and B, Percentage of low (<1 weekly admission per 100 beds) and high (≥15 weekly admission per 100 beds) COVID-19 admission rates, respectively. All distributions were calculated using hospital-level weights equal to the number of admissions in 2019. Florida is not shown due to data disclosure restrictions. Data were obtained from the Agency for Healthcare Research and Quality Healthcare Cost and Utilization Project State Inpatient Databases (2019-2020) for 45 US states.

Figure 2 presents the proportion of hospital-weeks with low and high COVID-19 admissions by state (full distribution presented in eTable 3 in Supplement 1). The proportion of hospital-weeks with low COVID-19 admissions varied substantially across the US, ranging from 6.1% in Nevada to 83.4% in Vermont. States where the proportion of hospital-weeks with low COVID-19 admissions was greater than 50.0% tended to be less densely populated. States with the lowest proportion of hospital-weeks with low COVID-19 admissions were concentrated in the Southwest and Southeast.

The proportion of hospital-weeks with high COVID-19 admissions also varied substantially, ranging from less than 1.0% in Maine, New Hampshire, and Vermont to 17.1% in Nevada. The proportion of hospital-weeks with high COVID-19 admissions was low in some rural states, like Maine (0.1%), but not others, like Montana (9.9%) and North Dakota (12.7%). States with the highest proportion of hospital-weeks with high COVID-19 admissions were concentrated in the Southwest, but also included New Jersey (13.8%), Illinois (12.9%), and North Dakota (12.7%).

### Association Between COVID-19 Exposure and Occupancy

Table 1 presents changes in inpatient occupancy during weeks with low and high COVID-19 admission rates. During weeks with low COVID-19 admissions, inpatient occupancy decreased by 9.3 percentage points per 100 beds (12.7% [95% CI, 12.1% to 13.4%] relative to the mean [SE] of 73.4 [0.5] in 2019). The percentage-point decrease in occupancy early in the pandemic (11.6 [15.8%]) was more pronounced than that in the second half of 2020 (5.2 [7.2%]). The largest percentage-point decrease was 10.3 (13.9%) in other metropolitan counties; the percentage-point decrease was similar in large metropolitan counties (9.2 [11.8%]), whereas the percentage-point decrease was largest in rural counties (5.6 [15.3%]). The decrease was similar in size when weighted by different races or ethnicities.

**Table 1.** Changes in Inpatient Occupancy During Low and High COVID-19 Admissions for 45 US States^a^

Sample	Inpatient occupancy per 100 beds		
	2019, Mean (SE)^b^	Percentage-point change (95% CI)^c^	
		Low COVID-19 admissions	High COVID-19 admissions
All hospitals	73.4 (0.5)	−9.3 (−9.8 to −8.9)	5.8 (5.0 to 6.6)
Time period			
Weeks 11-26	73.4 (0.5)	−11.6 (−12.0 to −11.2)	−1.1 (−2.5 to 0.3)
Weeks 27-48	72.2 (0.5)	−5.2 (−6.0 to −4.5)	9.1 (8.4 to 9.7)
Hospital location			
Metropolitan, large central	77.8 (1.0)	−9.2 (−10.0 to −8.3)	4.5 (2.8 to 6.1)
Metropolitan, other	74.3 (0.6)	−10.3 (−11.0 to −9.7)	6.0 (5.1 to 6.9)
Micropolitan	51.9 (1.2)	−7.4 (−8.1 to −6.7)	10.1 (8.5 to 11.8)
Rural, noncore	36.5 (1.5)	−5.6 (−6.3 to −5.0)	10.0 (6.2 to 13.7)
All hospitals, weighted by admission by race and ethnicity^d^			
Hispanic	75.1 (0.9)	−9.9 (−10.5 to −9.2)	6.7 (5.5 to 8.0)
Non-Hispanic Black	75.9 (0.7)	−9.3 (−10.0 to −8.6)	4.7 (3.4 to 6.0)
Non-Hispanic White	72.6 (0.5)	−9.4 (−9.8 to −8.9)	5.8 (5.0 to 6.6)
Other^e^	75.9 (0.8)	−9.6 (−10.3 to −8.9)	4.4 (2.9 to 5.8)

^a^ Data are from the Agency for Healthcare Research and Quality Healthcare Cost and Utilization Project State Inpatient Databases (2019-2020) for 45 US states.

^b^ Means and regressions were weighted by the number of admissions for each hospital in 2019.

^c^ Changes compared outcomes for each hospital-week in weeks 11 to 48 of 2020 to the corresponding hospital-week in 2019 (ie, regression models included hospital-week fixed effects). Low COVID-19 admissions correspond to less than 1 weekly COVID-19 admission per 100 beds, whereas high COVID-19 admissions correspond to 15 or more weekly COVID-19 admissions per 100 beds.

^d^ Means and regressions weighted by number of admissions in 2019 by race and ethnicity. Analyses related to patient race and ethnicity excluded 5 states that had more than 5% of these data missing (Louisiana, Montana, North Dakota, Nebraska, and West Virginia).

^e^ Includes non-Hispanic American Indian or Alaska Native, Asian or Pacific Islander, or multiple races or ethnicities.

During weeks with high COVID-19 admissions, inpatient occupancy increased by 5.8 percentage points per 100 beds (7.9% [95% CI, 6.8% to 9.0%]). The estimated change early in the pandemic was small and not statistically significant; in the second half of 2020, occupancy increased by 9.1 percentage points (12.6%). Occupancy increased by 4.5 percentage points (5.8%) in large metropolitan hospitals vs 10.0 percentage points (27.4%) in rural hospitals. The increase was similar in size when weighted by different races and ethnicities. As shown in the full results (eTable 4 in Supplement 1), occupancy gradually increased with the COVID-19 admission rate. Regressions estimated for inpatient occupancy using the subsample of 33 states with ICU data (eTable 5 in Supplement 1) were very similar to our full sample of 45 states.

Table 2 presents changes in ICU occupancy during weeks with low and high COVID-19 admissions (complete results are provided in eTable 6 in Supplement 1). During weeks with low COVID-19 admissions, ICU occupancy decreased by 3.1 percentage points per 100 beds (5.4% [95% CI, 3.8% to 7.0%]), reflecting a decrease of 4.9 percentage points (8.5%) early in the pandemic and a small, nonsignificant change during the second half of 2020. In absolute terms, the size of the decrease was similar for metropolitan and rural hospitals, but relative to the mean it was greatest for rural hospitals: ICU occupancy in rural hospitals decreased by 3.1 percentage points (10.2%). The decrease was similar in size when weighted by different races or ethnicities.

**Table 2.** Changes in ICU Occupancy During Low and High COVID-19 Admissions for 33 US States^a^

Characteristic	ICU occupancy per 100 beds		
	2019, mean (SE)^b^	Percentage-point change (95% CI)^c^	
		Low COVID-19 admissions	High COVID-19 admissions
All hospitals	57.4 (0.7)	−3.1 (−4.0 to −2.2)	38.9 (34.7 to 43.2)
Time period			
Weeks 11-26	57.6 (0.7)	−4.9 (−5.9 to −3.9)	53.4 (45.4 to 61.3)
Weeks 27-48	55.8 (0.7)	−0.1 (−1.6 to 1.3)	29.7 (26.0 to 33.3)
Hospital location			
Metropolitan, large central	63.7 (1.1)	−2.2 (−5.0 to 0.7)	41.6 (33.8 to 49.3)
Metropolitan, other	56.5 (0.8)	−3.9 (−4.9 to −2.8)	39.4 (33.8 to 45.0)
Micropolitan	42.7 (1.1)	−1.3 (−2.7 to 0.1)	25.3 (20.0 to 30.5)
Rural, noncore	30.2 (1.9)	−3.1 (−4.3 to −2.0)	15.1 (9.3 to 20.9)
All hospitals, weighted by admission by race and ethnicity^d^			
Hispanic	59.7 (1.2)	−1.8 (−3.8 to 0.2)	53.7 (43.8 to 63.6)
Non-Hispanic Black	60.2 (0.8)	−2.8 (−4.5 to −1.1)	37.8 (33.3 to 42.4)
Non-Hispanic White	56.2 (0.7)	−3.5 (−4.4 to −2.6)	34.7 (30.7 to 38.7)
Other^e^	58.7 (1.3)	−2.5 (−4.0 to −1.0)	47.3 (40.8 to 53.8)

Abbreviation: ICU, intensive care unit.

^a^ Data are from the Agency for Healthcare Research and Quality Healthcare Cost and Utilization Project State Inpatient Databases (2019-2020) for 33 US states.

^b^ Means and regressions were weighted by the number of admissions for each hospital in 2019.

^c^ Changes compared outcomes for each hospital-week in weeks 11 to 48 of 2020 to the corresponding hospital-week in 2019 (ie, regression models included hospital-week fixed effects). Low COVID-19 admission rates correspond to less than 1 weekly COVID-19 admission per 100 beds, whereas high COVID-19 admission rates correspond to 15 or more weekly COVID-19 admissions per 100 beds.

^d^ Means and regressions weighted by number of admissions in 2019 by race and ethnicity. Analyses related to patient race and ethnicity excluded 5 states that had more than 5% of these data missing (Louisiana, Montana, North Dakota, Nebraska, and West Virginia).

^e^ Other includes non-Hispanic American Indian or Alaska Native, Asian or Pacific Islander, or multiple races or ethnicities.

During weeks with high COVID-19 admissions, ICU occupancy increased substantially by 38.9 percentage points (67.8% relative to the mean [95% CI, 60.5% to 75.3%]). The increase was largest early in the pandemic at 53.4 percentage points (92.7%), decreasing to 29.7 percentage points (53.2%) later in the year. This increase was greatest in large metropolitan hospitals, with ICU occupancy increasing by 41.6 percentage points (65.3%), although all hospitals experienced substantial increases. For example, ICU occupancy rose by 15.1 percentage points (50.0%) in rural hospitals. When weighting by race and ethnicity, the change in ICU occupancy was largest for Hispanic individuals, increasing by 53.7 percentage points (89.9%). eTable 7 in Supplement 1 shows that there were similarly sized increases in the percentage of hospitals with inpatient and ICU occupancy rates greater than 75.0% and 90.0% during weeks with high COVID-19 admissions.

Table 3 presents changes in occupancy by hospital service line during weeks with low and high COVID-19 admissions (complete results are provided in eTable 8 in Supplement 1). For maternal, mental health and substance use disorders, injury, and surgical service lines, occupancy rates decreased in 2020 relative to 2019. Relative to the mean, the decreases were the most pronounced for surgical occupancy. For all service lines, the largest decreases occurred during weeks with high COVID-19 admissions early in the PHE, ranging from a decrease of 1.1 percentage points (9.9% [95% CI, 7.2% to 11.7%]) for maternal patients to 8.5 percentage points (43.1% [95% CI, 38.6% to 47.2%]) for surgical patients. Early in the PHE, decreases during weeks with low COVID-19 admissions ranged from a decline of 0.6 percentage points (5.4%) for maternal patients to 3.7 percentage points (18.8%) for surgical patients. During the second half of 2020, decreases moderated, with surgical patients declining by 1.2 percentage points (6.1%) during weeks with low COVID-19 admissions and 2.3 percentage points (11.7%) in weeks with high COVID-19 admissions.

**Table 3.** Changes in Occupancy by Service Line During Low and High COVID-19 Admissions for 45 US States^a^

Service line	Occupancy per 100 beds									
	2019, Mean (SE)^b^	2020 with COVID-19, %	Low COVID-19 admissions				High COVID-19 admissions			
			Weeks 11-26		Weeks 27-48		Weeks 11-26		Weeks 27-48	
			Percentage-point change (95% CI)	Change relative to mean, %^c^	Percentage-point change (95% CI)	Change relative to mean, %	Percentage-point change (95% CI)	Change relative to mean, %	Percentage-point change (95% CI)	Change relative to mean, %
Maternal	11.1 (0.2)	1.1 (0.1)	−0.6 (−0.7 to −0.5)	−5.4	−0.9 (−1.1 to −0.7)	−8.1	−1.1 (−1.3 to −0.8)	−9.9	−0.7 (−0.9 to −0.5)	−6.3
Mental health and substance use disorder	5.7 (0.2)	1.5 (0.1)	−0.7 (−0.8 to −0.6)	−12.3	−0.4 (−0.7 to −0.2)	−7.0	−2.3 (−2.8 to −1.9)	−40.4	−0.3 (−0.4 to −0.2)	−5.3
Injury	4.2 (0.1)	2.0 (0)	−0.4 (−0.4 to −0.3)	−9.5	0 (−0.1 to 0.1)	0	−1.2 (−1.3 to −1.0)	−28.6	−0.2 (−0.3 to −0.1)	−4.8
Surgery	19.7 (0.2)	4.2 (0.1)	−3.7 (−3.9 to −3.5)	−18.8	−1.2 (−1.4 to −1.0)	−6.1	−8.5 (−9.3 to −7.6)	−43.1	−2.3 (−2.5 to −2.0)	−11.7
Medical	39.4 (0.3)	11.8 (0.1)	−7.5 (−7.7 to −7.2)	−19.0	−3.3 (−3.7 to −2.9)	−8.4	10.7 (9.5 to 11.9)	27.2	12.4 (11.9 to 13.0)	31.5

^a^ Data are from the Agency for Healthcare Research and Quality Healthcare Cost and Utilization Project State Inpatient Databases (2019-2020) for 45 US states.

^b^ All results were weighted by the number of admissions for each hospital in 2019.

^c^ Changes compared outcomes for each hospital-week in weeks 11 to 48 of 2020 to the corresponding hospital-week in 2019 (ie, regression models included hospital-week fixed effects). Low COVID-19 admission rates correspond to less than 1 weekly COVID-19 admission per 100 beds, whereas high COVID-19 admission rates correspond to 15 or more weekly COVID-19 admissions per 100 beds.

Most patients with COVID-19 were treated by the medical service line. During weeks with low COVID-19 admissions, occupancy also decreased for medical patients; it declined by 7.5 percentage points (19.0% [95% CI, 18.3%-19.5%]) early in the PHE and by 3.3 (8.4% [95% CI, 7.4%-9.4%]) in the second half of 2020. In contrast to the other service lines, occupancy for medical patients increased during weeks with high COVID-19 admissions; it increased by 10.7 percentage points (27.2%) early in the pandemic and by 12.4 percentage points (31.5%) in the second half of 2020.

## Discussion

Using data on hospitalizations in 45 states, we observed that between March 11 and November 26, 2020, weekly COVID-19 admission rates were less than 4 per 100 beds for 63.9% of hospital-weeks and at least 10 per 100 beds in 15.9% of hospital-weeks. During weeks with high COVID-19 admissions (≥15), inpatient occupancy increased by 7.9% and ICU occupancy increased by 67.8%. The increase in ICU occupancy was greatest when we weighted the analysis to reflect the experiences of Hispanic patients.

Previous work using data from 201 hospitals documented a 43% decrease in non–COVID-19 medical admissions and a 34% decrease in all medical admissions for April 2020 compared with April 2019.^5^ Our analysis of all medical and nonmedical admissions in 3960 hospitals showed that decreases in inpatient occupancy during the first half of 2020 were concentrated in hospitals with low COVID-19 admission rates. Occupancy rates for many types of service lines, such as surgery, also decreased in hospitals with high COVID-19 admission rates; however, total inpatient occupancy increased, especially in ICUs.

Multiple factors contributed to variations in hospital occupancy during 2020. Occupancy reductions in hospitals with low COVID-19 admission rates were likely driven by patients who feared contagion, policies to defer elective procedures, and stay-at-home orders.^28,29^ In the second half of 2020, we observed smaller occupancy reductions, possibly because these factors subsided. During this period, we also observed smaller increases in ICU occupancy during surges, possibly because of improvements in COVID-19 treatment.^30^

Changes in occupancy during COVID-19 may have affected patient care. Large increases in ICU occupancy may have strained ICU staff, possibly reducing quality of care for critically ill patients. This result is in line with increased in-hospital mortality during COVID-19 surges, documented by previous studies.^5,6,7,8,9,10,11,12^ In contrast, in our study, COVID-19 surges reduced occupancy for surgeries, injuries, and deliveries, likely reflecting deferred care for some conditions. Emerging evidence suggests that deferrals of care during the pandemic also increased mortality.^31,32^

Changes in occupancy also had operational consequences for hospitals. During the 63.9% of hospital-weeks when COVID-19 admission rates were less than 4 per 100 beds, occupancy rates were substantially lower than in 2019. These low occupancy rates contributed to financial losses for hospitals, evidenced by decreases in operating margins, net patient revenues, and net operating income excluding COVID-19 relief funding in 2020.^13,14,15,16,17^ Rural hospitals were most likely to experience low COVID-19 admission rates and had the largest decreases in occupancy relative to the mean.

Transitions between weeks with low and high occupancy may have also posed operational challenges. Early in the pandemic, ICU occupancy rates decreased by 8.5% during weeks with low COVID-19 admissions and increased by 92.7% during weeks with high COVID-19 admissions. Such fluctuations likely contributed to increased reliance on travel nursing, which is more expensive for hospitals and may pose challenges integrating temporary employees.^33,34,35,36,37^ Rural hospitals also experienced large swings in overall inpatient occupancy, with rates declining by 15.3% during weeks with low COVID-19 admissions and increasing by 27.4% during weeks with high COVID-19 admissions.

Our findings suggest that COVID-19 surges posed different challenges for metropolitan hospitals. These hospitals had higher baseline ICU occupancy rates; thus, COVID-19 surges often increased occupancy to 100% of beds or more. For example, ICU occupancy in large metropolitan hospitals averaged 63.9% in 2019 and increased by 42 percentage points during weeks with high COVID-19 admissions. As a result, many metropolitan hospitals converted other critical care units to ICUs and delayed surgeries that required recovery in ICUs.^18,22,38,39,40,41^

Our results also suggest that Hispanic patients were less likely than White patients to be admitted to hospitals with low COVID-19 burden, mirroring evidence from September 2020 to November 2021 that hospitals disproportionately serving Black patients were more likely to experience ICU occupancy greater than 90.0%.^42^ Thus, at different points in the pandemic, both Black and Hispanic patients were more likely to be admitted to capacity-strained hospitals than White patients. Moreover, using data from 2015 to 2016, Singh et al^43^ found that mortality increased more for Black patients than White patients when hospital occupancy approached capacity. This collection of evidence suggests that hospital care may have contributed to the disproportionate increases in Black and Hispanic mortality observed during the COVID-19 pandemic.^44^

Our results highlight the importance of policies that provided financial aid or deployed resources (including equipment and staff) to hospitals during the pandemic. Differences in the incidence of COVID-19 surges across states and over time also emphasize the importance of data-gathering programs such as the Unified Hospital Data Surveillance System, which was implemented during the pandemic to gather daily data on hospital strain and helped target distribution of aid.^45^ These differences also emphasize the importance of flexible recommendations for hospitals that reflect local conditions. During periods with low COVID-19 admission rates (which represented over half of hospital-weeks in 2020 for states such as Vermont), guidance to reduce elective admissions may have caused deferrals in needed care and harmed hospital finances, although the risk of COVID-19 contagion was small.

### Limitations

Our study has several limitations. First, COVID-19 cases may have been undercounted, especially during the first half of 2020, because tests were not widely available until the summer and *ICD-10-CM* codes for COVID-19 were not introduced until April 1, 2020. To address these issues, we identified COVID-19 cases prior to April following coding recommendations,^24^ and we split the sample into early and late 2020. Nonetheless, we caution that surges during the early pandemic may differ from surges during the second half of 2020 because of differences in the accuracy of identifying COVID-19. Second, the analysis of ICU occupancy was only possible for 33 states that reported revenue code data. Regressions estimated for inpatient occupancy using the subsample of 33 states were very similar to our full sample of 45 states, suggesting that our ICU occupancy results may also extend to a broader set of states. Finally, factors unrelated to COVID-19 may have contributed to changes in occupancy during 2020. The effect of such factors is probably relatively small: in our full set of coefficient estimates, occupancy rates during weeks 1 to 10 of 2020 were very similar to weeks 1 to 10 of 2019.

## Conclusions

In this cross-sectional study, hospital occupancy decreased in 2020 during weeks with low COVID-19 admissions and increased during weeks with high COVID-19 admissions. Surges in COVID-19 strained ICUs and were associated with large decreases in surgical occupancy. Research is needed to further assess associations between these occupancy fluctuations and quality of care and hospital financial stability.

## Notes

Supplement 1. **eTable 1.** States Included in Each Analysis **eTable 2. **Percent Distribution of Hospital-Weeks by COVID-19 Admission Rate per 100 Inpatient Beds and Hospital Type, 2020 Weeks 11 to 48 **eTable 3.** Percent Distribution of Hospital-Weeks by COVID-19 Admission Rate per 100 Inpatient Beds and State, 2020 Weeks 11 to 48 **eTable 4. **Changes in Inpatient Occupancy During 2020, Full Regression Results **eTable 5.** Changes in Inpatient Occupancy During Low and High COVID-19 Admissions, Sample of 33 States With Data on Intensive Care Unit Admissions **eTable 6.** Changes in Intensive Care Unit (ICU) Occupancy During 2020, Full Regression Results, Sample of 33 States With Data on ICU Admissions **eTable 7.** Changes in Occupancy Rates and Thresholds During Low and High COVID-19 Admissions **eTable 8. **Changes in Occupancy by Service Line During 2020, Full Regression Results **eFigure 1.** Distribution of Duration of Periods With High COVID-19 Admissions **eFigure 2.** Percentage of Hospitals With High COVID-19 Admission Rates by Week in 2020

Supplement 2.
Data Sharing Statement
