# Supplementary material for: COVID-19 Admission Rates and Changes in US Hospital Inpatient and Intensive Care Unit Occupancy
Source: JAMA Health Forum. 2023 Dec 1;4(12):e234206. doi: 10.1001/jamahealthforum.2023.4206 (PMC10692846; doi:10.1001/jamahealthforum.2023.4206)
Supplement: Supplement 1. — eTable 1. States Included in Each Analysis eTable 2. Percent Distribution of Hospital-Weeks by COVID-19 Admission Rate per 100 Inpatient Beds and Hospital Type, 2020 Weeks 11 to 48 eTable 3. Percent Distribution of Hospital-Weeks by COVID-19 Admission Rate per 100 Inpatient Beds and State, 2020 Weeks 11 to 48 eTable 4. Changes in Inpatient Occupancy During 2020, Full Regression Results eTable 5. Changes in Inpatient Occupancy During Low and High COVID-19 Admissions, Sample of 33 States With Data on Intensive Care Unit Admissions eTable 6. Changes in Intensive Care Unit (ICU) Occupancy During 2020, Full Regression Results, Sample of 33 States With Data on ICU Admissions eTable 7. Changes in Occupancy Rates and Thresholds During Low and High COVID-19 Admissions eTable 8. Changes in Occupancy by Service Line During 2020, Full Regression Results eFigure 1. Distribution of Duration of Periods With High COVID-19 Admissions eFigure 2. Percentage of Hospitals With High COVID-19 Admission Rates by Week in 2020 [file jamahealthforum-e234206-s001.pdf]

Supplementary Online Content

Meille G, Decker SL, Owens PL, Selden TM. COVID-19 admission rates and changes in US hospital inpatient and intensive care unit occupancy. *JAMA Health Forum*. 2023;4(12):e234206. doi:10.1001/jamahealthforum.2023.4206

eTable 1. States Included in Each Analysis

eTable 2. Percent Distribution of Hospital-Weeks by COVID-19 Admission Rate per 100 Inpatient Beds and Hospital Type, 2020 Weeks 11 to 48

eTable 3. Percent Distribution of Hospital-Weeks by COVID-19 Admission Rate per 100 Inpatient Beds and State, 2020 Weeks 11 to 48

eTable 4. Changes in Inpatient Occupancy During 2020, Full Regression Results

eTable 5. Changes in Inpatient Occupancy During Low and High COVID-19 Admissions, Sample of 33 States With Data on Intensive Care Unit Admissions

eTable 6. Changes in Intensive Care Unit (ICU) Occupancy During 2020, Full Regression Results, Sample of 33 States With Data on ICU Admissions

eTable 7. Changes in Occupancy Rates and Thresholds During Low and High COVID-19 Admissions

eTable 8. Changes in Occupancy by Service Line During 2020, Full Regression Results

eFigure 1. Distribution of Duration of Periods With High COVID-19 Admissions

eFigure 2. Percentage of Hospitals With High COVID-19 Admission Rates by Week in 2020

This supplementary material has been provided by the authors to give readers additional information about their work.

© 2023 Meille G et al. *JAMA Health Forum*.

eTable 1. States Included in Each Analysis

Analysis	Number of States	List of States
Primary analysis	45	AK, AR, AZ, CA, DC, DE, FL, GA, HI, IL, IN, IA, KS, KY, LA, MA, MD, ME, MI, MN, MO, MS, MT, NC, ND, NE, NH, NJ, NM, NV, NY, OH, OK, OR, RI, SC, SD, TN, TX, UT, VA, VT, WI, WV, WY
ICU analysis[a]	33	AK, AR, DE, GA, IL, KS, KY, LA, MA, MD, ME, MN, MO, MT, NC, ND, NE, NH, NJ, NM, NV, NY, OK, OR, SC, TN, TX, UT, VA, VT, WI, WV, WY
Only primary analysis	12	AZ, CA, DC, FL, HI, IN, IA, MI, MS, OH, RI, SD
No analyses[b]	6	AL, CO, CT, ID, PA, WA

[a] States were included in the ICU analysis if they reported revenue codes to the Agency for Healthcare Research and Quality, Healthcare Cost and Utilization Project (HCUP) State Inpatient Databases (SID) for 2019-2020

[b] States were not included in analyses if they did not provide data to HCUP or they did not provide exact admission dates.

© 2023 Meille G et al. *JAMA Health Forum*.

eTable 2. Percent Distribution of Hospital-Weeks by COVID-19 Admission Rate per 100 Inpatient Beds and Hospital Type, 2020 Weeks 11 to 48[a,b,c]

	COVID-19 rate <1	COVID-19 rate 1-4	COVID-19 rate 5-10	COVID-19 rate 10-14	COVID-19 rate 15+	Number of Hospitals
All hospitals						
All hospitals	20.0	43.9	20.2	8.0	7.9	3,960
Time period						
Weeks 11-26	30.5	44.0	14.3	5.0	6.1	3,960
Weeks 27-48	12.3	43.8	24.5	10.1	9.2	3,960
Hospital location						
Metro, large central	14.5	48.4	21.4	8.0	7.7	751
Metro, other	20.1	43.2	20.3	8.2	8.2	1,624
Micropolitan	38.0	31.1	16.2	7.4	7.3	641
Rural, non-core	59.2	19.3	9.9	4.9	6.7	944
All hospitals, weighted by admissions by race/ethnicity[c]						
Hispanic	15.1	41.9	23.2	9.4	10.4	3,693
Non-Hispanic Black	13.9	46.7	22.5	8.7	8.1	3,693
Non-Hispanic White	22.2	43.5	19.1	7.7	7.5	3,693
Other race[e]	18.2	45.8	20.4	7.3	8.4	3,693

[a] Data are from the Agency for Healthcare Research and Quality Healthcare Cost and Utilization Project State Inpatient Databases (2019-2020) for 45 US states.
[b] All distributions calculated using hospital level weights equal to the number of admissions in 2019.
[c] For each panel the reference category is the first one listed. For all other categories, the difference between their distribution and that of the reference category is statistically significant at the 1% level.
[d] Distributions weighted by number of admissions in 2019 by race/ethnicity. Analyses related to patient race and ethnicity excluded 5 states that had more than 5% of these data missing (Louisiana, Montana, North Dakota, Nebraska, and West Virginia).
[e] Other includes non-Hispanic American Indian or Alaska Native, Asian or Pacific Islander, or multiple races or ethnicities.

© 2023 Meille G et al. *JAMA Health Forum*.

eTable 3. Percent Distribution of Hospital-Weeks by COVID-19 Admission Rate per 100 Inpatient Beds and State, 2020 Weeks 11 to 48[a,b]

State	COVID-19 rate <1	COVID-19 rate 1-4	COVID-19 rate 5-10	COVID-19 rate 10-14	COVID-19 rate 15+	Number of Hospitals
AK	50.5	30.8	11.9	4.9	1.8	21
AR	30.4	35.7	23.8	5.9	4.2	74
AZ	13.8	45.0	19.9	8.9	12.4	64
CA	15.9	46.0	25.1	7.6	5.4	331
DC	10.1	60.1	15.9	9.4	4.5	7
DE	8.7	57.8	22.5	6.5	4.4	6
FL[c]	sup.	sup.	sup.	sup.	sup.	179
GA	7.5	36.2	33.1	14.6	8.7	129
HI	52.8	31.4	10.6	4.4	0.9	21
IA	21.5	45.3	17.9	7.7	7.6	115
IL	13.3	43.0	18.6	12.1	12.9	175
IN	16.5	50.8	18.1	7.1	7.5	115
KS	28.9	46.7	13.5	4.7	6.3	118
KY	28.3	47.6	15.2	5.8	3.1	92
LA	9.5	44.7	29.4	10.2	6.3	78
MA	22.4	44.8	15.2	7.6	10.1	61
MD	7.8	44.5	27.3	10.3	10.0	45
ME	67.3	29.7	2.5	0.4	0.1	33
MI	23.4	47.4	11.8	7.2	10.2	122
MN	24.6	42.6	19.0	5.7	8.2	125
MO	25.8	42.7	17.5	7.9	6.1	108
MS	11.6	41.4	32.2	8.9	6.0	88
MT	48.6	20.0	14.6	7.0	9.9	42
NC	15.1	52.2	24.6	6.2	1.8	102
ND	31.1	37.2	11.3	7.6	12.7	16
NE	25.6	45.7	16.2	5.3	7.2	86
NH	55.1	35.2	8.3	1.1	0.3	26
NJ	16.7	44.6	15.2	9.7	13.8	64
NM	20.9	46.0	14.1	5.5	13.6	35
NV	6.1	37.4	24.0	15.5	17.1	34
NY	27.9	46.8	10.8	3.6	10.9	158
OH	24.2	51.0	14.0	4.3	6.5	155
OK	30.5	35.5	23.9	5.8	4.4	111
OR	47.6	42.9	6.4	2.0	1.0	60
RI	16.6	46.8	17.6	9.9	9.1	10
SC	15.4	42.5	26.4	10.9	4.8	59
SD	27.1	38.3	13.9	10.5	10.2	50
TN	21.4	41.6	22.8	8.5	5.7	98
TX	16.7	36.0	24.9	10.9	11.4	407
UT	20.6	43.6	23.4	6.9	5.4	48
VA	15.1	57.4	18.3	5.6	3.5	81
VT	83.4	15.6	0.9	0.1	0.1	14
WI	26.3	40.0	15.4	8.0	10.3	129
WV	51.2	34.6	11.8	1.4	1.0	45
WY	69.4	17.1	6.1	2.2	5.2	23

[a] Data are from the Agency for Healthcare Research and Quality Healthcare Cost and Utilization Project State Inpatient Databases (2019-2020) for 45 US states.
[b] All distributions calculated using hospital level weights equal to the number of admissions in 2019.
[c] Distribution in Florida suppressed (sup.) because of disclosure restrictions.

© 2023 Meille G et al. *JAMA Health Forum*.

eTable 4. Changes in Inpatient Occupancy During 2020, Full Regression Results

Panel A. All hospitals, weeks 11-26, and weeks 27-48[a,b]

	All hospitals	Weeks 11-26	Weeks 27-48
	Percentage-point change in occupancy per 100 beds (95% CI)		
Weeks 1-10*COVID[c]<1	0.6 (0.3 to 0.9)	N/A	N/A
Weeks 1-10*COVID[c]≥1	0.3 (-1.0 to 1.6)	N/A	N/A
Weeks 11-48*COVID[c]<1	-9.3 (-9.8 to -8.9)	-11.6 (-12.0 to -11.2)	-5.2 (-6.0 to -4.5)
Weeks 11-48*COVID[c] 1-4	-6.7 (-7.1 to -6.3)	-12.2 (-12.6 to -11.7)	-2.7 (-3.2 to -2.3)
Weeks 11-48*COVID[c] 5-10	-2.8 (-3.2 to -2.3)	-10.0 (-10.7 to -9.3)	0.3 (-0.1 to 0.7)
Weeks 11-48*COVID[c] 10-15	0.3 (-0.2 to 0.9)	-7.7 (-8.9 to -6.6)	3.3 (2.8 to 3.7)
Weeks 11-48*COVID[c] 15+	5.8 (5.0 to 6.6)	-1.1 (-2.5 to 0.3)	9.1 (8.4 to 9.7)
Mean of occupancy in 2019	73.4	73.4	72.2
N	374880	124960	171820

[a] Data are from the Agency for Healthcare Research and Quality Healthcare Cost and Utilization Project State Inpatient Databases (2019-2020) for 45 US states.
[b] Regressions compared outcomes for each hospital-week of 2020 to the corresponding hospital-week in 2019, i.e., regression models included hospital-week fixed effects. Means and regressions were weighted by the number of admissions for each hospital in 2019.
[c] COVID denotes weekly COVID-19 admissions per 100 beds.

© 2023 Meille G et al. *JAMA Health Forum*.

Panel B. Urbanicity of hospital county[a,b]

	Metro, large central	Metro, other	Micropolitan	Rural, non-core
	Percentage-point change in occupancy per 100 beds (95% CI)			
Weeks 1-10*COVID[c]<1	0.9<br>(0.4 to 1.5)	0.6<br>(0.1 to 1.0)	-0.7<br>(-1.3 to -0.0)	-0.9<br>(-2.1 to 0.4)
Weeks 1-10*COVID[c]≥1	-1.3<br>(-3.1 to 0.6)	1.2<br>(-0.6 to 2.9)	1.6<br>(-2.7 to 5.9)	1.9<br>(-6.8 to 10.6)
Weeks 11-48*COVID[c]<1	-9.2<br>(-10.0 to -8.3)	-10.3<br>(-11.0 to -9.7)	-7.4<br>(-8.1 to -6.7)	-5.6<br>(-6.3 to -5.0)
Weeks 11-48*COVID[c] 1-4	-7.5<br>(-8.2 to -6.9)	-6.3<br>(-6.7 to -5.9)	-4.3<br>(-5.1 to -3.5)	-4.3<br>(-6.1 to -2.6)
Weeks 11-48*COVID[c] 5-10	-4.4<br>(-5.1 to -3.7)	-1.8<br>(-2.4 to -1.2)	0.0<br>(-0.9 to 0.8)	-1.6<br>(-3.9 to 0.8)
Weeks 11-48*COVID[c] 10-15	-1.6<br>(-2.6 to -0.5)	1.2<br>(0.5 to 1.9)	4.2<br>(3.2 to 5.1)	0.9<br>(-2.2 to 4.0)
Weeks 11-48*COVID[c] 15+	4.5<br>(2.8 to 6.1)	6.0<br>(5.1 to 6.9)	10.1<br>(8.5 to 11.8)	10.0<br>(6.2 to 13.7)
Mean of occupancy in 2019	77.8	74.3	51.9	36.5
N	70848	153792	60864	89376

[a] Data are from the Agency for Healthcare Research and Quality Healthcare Cost and Utilization Project State Inpatient Databases (2019-2020) for 45 US states.

[b] Regressions compared outcomes for each hospital-week of 2020 to the corresponding hospital-week in 2019, i.e., regression models included hospital-week fixed effects. Means and regressions were weighted by the number of admissions for each hospital in 2019.

[c] COVID denotes weekly COVID-19 admissions per 100 beds.

© 2023 Meille G et al. *JAMA Health Forum*.

Panel C. All hospitals, weighted by admissions by race/ethnicity[a,b,d]

	Hispanic	Non-Hispanic Black	Non-Hispanic White	Other[e]
	Percentage-point change in occupancy per 100 beds (95% CI)			
Weeks 1-10*COVID[c]<1	0.9 (0.4 to 1.4)	0.7 (0.4 to 1.0)	1.0 (0.6 to 1.5)	0.7 (0.3 to 1.2)
Weeks 1-10*COVID[c]≥1	0.5 (-1.4 to 2.3)	0.3 (-1.3 to 1.9)	-0.3 (-1.6 to 0.9)	-1.1 (-2.5 to 0.4)
Weeks 11-48*COVID[c]<1	-9.3 (-10.0 to -8.6)	-9.4 (-9.8 to -8.9)	-9.9 (-10.5 to -9.2)	-9.6 (-10.3 to -8.9)
Weeks 11-48*COVID[c] 1-4	-6.8 (-7.4 to -6.3)	-6.6 (-6.9 to -6.2)	-7.5 (-8.2 to -6.9)	-7.5 (-8.3 to -6.7)
Weeks 11-48*COVID[c] 5-10	-3.0 (-3.6 to -2.3)	-2.3 (-2.7 to -1.9)	-4.1 (-4.8 to -3.4)	-4.3 (-5.1 to -3.5)
Weeks 11-48*COVID[c] 10-15	-0.3 (-1.1 to 0.5)	1.0 (0.4 to 1.5)	-0.6 (-1.5 to 0.2)	-1.4 (-2.3 to -0.4)
Weeks 11-48*COVID[c] 15+	4.7 (3.4 to 6.0)	5.8 (5.0 to 6.6)	6.7 (5.5 to 8.0)	4.4 (2.9 to 5.8)
Mean of occupancy in 2019	75.1	75.9	72.6	75.9
N	316800	347424	305088	317376

[a] Data are from the Agency for Healthcare Research and Quality Healthcare Cost and Utilization Project State Inpatient Databases (2019-2020) for 45 US states.

[b] Regressions compared outcomes for each hospital-week of 2020 to the corresponding hospital-week in 2019, i.e., regression models included hospital-week fixed effects. Means and regressions were weighted by the number of admissions for each hospital in 2019.

[c] COVID denotes weekly COVID-19 admissions per 100 beds.

[d] States without ICU data and states missing more than 5% of data on race/ethnicity excluded (Louisiana, Montana, North Dakota, Nebraska, and West Virginia).

© 2023 Meille G et al. *JAMA Health Forum*.

eTable 5. Changes in Inpatient Occupancy During Low and High COVID-19 Admissions, Sample of 33 States With Data on Intensive Care Unit Admissions[a,b,c]

	2019 occupancy (per 100 beds), mean	Change during low COVID-19[d]	Change during high COVID-19[e]
		Percentage-point change in occupancy per 100 beds (95% CI)	
**All hospitals**			
All hospitals	73.5	-9.2 (-9.8 to -8.6)	5.1 (4.1 to 6.2)
**Time period**			
Weeks 11-26	73.4	-11.5 (-12.1 to -11.0)	-0.9 (-2.5 to 0.6)
Weeks 27-48	72.5	-5.5 (-6.5 to -4.5)	9.0 (8.1 to 9.8)
**Hospital location**			
Metro, large central	79.9	-8.8 (-10.0 to -7.5)	3.9 (1.8 to 6.1)
Metro, other	74.1	-10.2 (-11.1 to -9.4)	5.3 (4.1 to 6.4)
Micropolitan	52.6	-7.3 (-8.1 to -6.4)	9.2 (7.9 to 10.6)
Rural, non-core	37.5	-5.7 (-6.5 to -4.9)	10.2 (5.5 to 14.9)
**All hospitals, weighted by admission by race/ethnicity[f]**			
Hispanic	74.9	-9.4 (-10.4 to -8.5)	5.3 (3.7 to 6.9)
Non-Hispanic Black	76.2	-9.1 (-10.1 to -8.2)	4.5 (3.0 to 6.0)
Non-Hispanic White	73.0	-9.3 (-9.9 to -8.7)	5.1 (4.1 to 6.1)
Other race[g]	76.4	-9.0 (-9.9 to -8.0)	4.1 (2.3 to 6.0)

[a] Data are from the Agency for Healthcare Research and Quality Healthcare Cost and Utilization Project State Inpatient Databases (2019-2020) for 33 US states.
[b] Changes compared outcomes for each hospital-week in weeks 11-48 of 2020 to the corresponding hospital-week in 2019, i.e., regression models included hospital-week fixed effects.
[c] Means and regressions were weighted by the number of admissions for each hospital in 2019.
[d] Low COVID-19 admission rate corresponds to less than 1 weekly COVID-19 admission per 100 beds.
[e] High COVID-19 admission rate corresponds to 15 or more weekly COVID-19 admissions per 100 beds.

© 2023 Meille G et al. *JAMA Health Forum*.

[f] Regressions weighted by number of admissions in 2019 by race/ethnicity. States with more than 5% of data on race/ethnicity excluded (Louisiana, Montana, North Dakota, Nebraska, and West Virginia).
[g] Other includes non-Hispanic American Indian or Alaska Native, Asian or Pacific Islander, or multiple races or ethnicities.

© 2023 Meille G et al. *JAMA Health Forum*.

eTable 6. Changes in Intensive Care Unit (ICU) Occupancy During 2020, Full Regression Results, Sample of 33 States With Data on ICU Admissions

Panel A. All hospitals, weeks 11-26, and weeks 27-48[a,c]

	All hospitals	Weeks 11-26	Weeks 27-48
	Percentage-point change in occupancy (95% CI)		
Weeks 1-10*COVID[c]<1	0.6<br>(-0.1 to 1.4)	N/A	N/A
Weeks 1-10*COVID[c]≥1	-0.6<br>(-3.3 to 2.1)	N/A	N/A
Weeks 11-48*COVID[c]<1	-3.1<br>(-4.0 to -2.2)	-4.9<br>(-5.9 to -3.9)	-0.1<br>(-1.6 to 1.3)
Weeks 11-48*COVID[c] 1-4	4.1<br>(3.1 to 5.1)	1.7<br>(0.6 to 2.8)	5.8<br>(4.5 to 7.0)
Weeks 11-48*COVID[c] 5-10	13.6<br>(12.2 to 15.1)	12.5<br>(10.3 to 14.8)	14.1<br>(12.6 to 15.7)
Weeks 11-48*COVID[c] 10-15	20.3<br>(18.5 to 22.1)	23.7<br>(20.2 to 27.3)	18.9<br>(17.1 to 20.8)
Weeks 11-48*COVID[c] 15+	38.9<br>(34.7 to 43.2)	53.4<br>(45.4 to 61.3)	29.7<br>(26.0 to 33.3)
Mean of occupancy in 2019	57.4	57.6	55.8
N	180672	60224	82808

[a] Data are from the Agency for Healthcare Research and Quality Healthcare Cost and Utilization Project State Inpatient Databases (2019-2020) for 33 US states.

[b] Regressions compared outcomes for each hospital-week of 2020 to the corresponding hospital-week in 2019, i.e., regression models included hospital-week fixed effects. Means and regressions were weighted by the number of admissions for each hospital in 2019.

[c] COVID denotes weekly COVID-19 admissions per 100 beds.

© 2023 Meille G et al. *JAMA Health Forum*.

Panel B. Urbanicity of hospital county[a,b]

	Metro, large central	Metro, other	Micropolitan	Rural, non-core
	Percentage-point change in occupancy per 100 beds (95% CI)			
Weeks 1-10*COVID[c]<1	0.1<br>(-1.3 to 1.5)	0.9<br>(-0.1 to 1.8)	1.0<br>(-0.4 to 2.4)	0.0<br>(-3.2 to 3.2)
Weeks 1-10*COVID[c]≥1	-2.6<br>(-8.4 to 3.2)	0.1<br>(-2.9 to 3.1)	2.2<br>(-5.7 to 10.1)	3.8<br>(-6.2 to 13.7)
Weeks 11-48*COVID[c]<1	-2.2<br>(-5.0 to 0.7)	-3.9<br>(-4.9 to -2.8)	-1.3<br>(-2.7 to 0.1)	-3.1<br>(-4.3 to -2.0)
Weeks 11-48*COVID[c] 1-4	3.7<br>(1.8 to 5.7)	4.1<br>(2.9 to 5.3)	6.3<br>(4.3 to 8.2)	2.1<br>(-0.5 to 4.8)
Weeks 11-48*COVID[c] 5-10	13.0<br>(10.0 to 16.1)	14.1<br>(12.4 to 15.8)	13.5<br>(10.0 to 17.0)	8.2<br>(4.6 to 11.9)
Weeks 11-48*COVID[c] 10-15	19.7<br>(16.3 to 23.1)	21.4<br>(19.0 to 23.7)	15.9<br>(12.8 to 19.0)	12.6<br>(5.4 to 19.8)
Weeks 11-48*COVID[c] 15+	41.6<br>(33.8 to 49.3)	39.4<br>(33.8 to 45.0)	25.3<br>(20.0 to 30.5)	15.1<br>(9.3 to 20.9)
Mean of occupancy in 2019	63.7	56.5	42.7	30.2
N	33504	86112	34848	26208

[a] Data are from the Agency for Healthcare Research and Quality Healthcare Cost and Utilization Project State Inpatient Databases (2019-2020) for 33 US states.
[b] Regressions compared outcomes for each hospital-week of 2020 to the corresponding hospital-week in 2019, i.e., regression models included hospital-week fixed effects. Means and regressions were weighted by the number of admissions for each hospital in 2019.
[c] COVID denotes weekly COVID-19 admissions per 100 beds.

© 2023 Meille G et al. *JAMA Health Forum*.

Panel C. All hospitals, weighted by admissions by race/ethnicity[a,b,d]

	Hispanic	Non-Hispanic Black	Non-Hispanic White	Other
	Percentage-point change in occupancy per 100 beds (95% CI)			
Weeks 1-10*COVID[c]<1	1.9<br>(0.5 to 3.4)	0.7<br>(-0.3 to 1.7)	0.8<br>(0.0 to 1.5)	-0.1<br>(-1.3 to 1.1)
Weeks 1-10*COVID[c]≥1	2.7<br>(-3.7 to 9.1)	-1.0<br>(-4.4 to 2.4)	-0.2<br>(-2.9 to 2.6)	-4.2<br>(-7.9 to -0.4)
Weeks 11-48*COVID[c]<1	-1.8<br>(-3.8 to 0.2)	-2.8<br>(-4.5 to -1.1)	-3.5<br>(-4.4 to -2.6)	-2.5<br>(-4.0 to -1.0)
Weeks 11-48*COVID[c] 1-4	4.4<br>(2.6 to 6.3)	5.0<br>(3.2 to 6.8)	3.6<br>(2.7 to 4.6)	3.0<br>(1.2 to 4.7)
Weeks 11-48*COVID[c] 5-10	17.3<br>(13.4 to 21.3)	14.2<br>(11.7 to 16.8)	12.8<br>(11.5 to 14.1)	13.6<br>(11.1 to 16.1)
Weeks 11-48*COVID[c] 10-15	25.1<br>(21.4 to 28.8)	19.6<br>(17.2 to 22.1)	19.5<br>(17.8 to 21.3)	21.9<br>(19.0 to 24.9)
Weeks 11-48*COVID[c] 15+	53.7<br>(43.8 to 63.6)	37.8<br>(33.3 to 42.4)	34.7<br>(30.7 to 38.7)	47.3<br>(40.8 to 53.8)
Mean of occupancy in 2019	59.7	60.2	56.2	57.8
N	150624	160608	164448	160416

[a] Data are from the Agency for Healthcare Research and Quality Healthcare Cost and Utilization Project State Inpatient Databases (2019-2020) for 33 US states.

[b] Regressions compared outcomes for each hospital-week of 2020 to the corresponding hospital-week in 2019, i.e., regression models included hospital-week fixed effects. Means and regressions were weighted by the number of admissions for each hospital in 2019.

[c] COVID denotes weekly COVID-19 admissions per 100 beds.

[d] States without ICU data and states missing more than 5% of data on race/ethnicity excluded (Louisiana, Montana, North Dakota, Nebraska, and West Virginia).

[e] Other includes non-Hispanic American Indian or Alaska Native, Asian or Pacific Islander, or multiple races or ethnicities.

© 2023 Meille G et al. *JAMA Health Forum*.

eTable 7. Changes in Occupancy Rates and Thresholds During Low and High COVID-19 Admissions

	2019, mean	Low COVID-19 admission rate[e]	High COVID-19 admission rate[f]
		Percentage-point change (95% CI)	
Inpatient occupancy[c]	73.4	-9.3 (-9.8 to -8.9)	5.8 (5.0 to 6.6)
Inpatient occupancy[c] ≥ 75%	49.5	-17.5 (-19.3 to -15.7)	10.3 (7.9 to 12.7)
Inpatient occupancy[c] ≥ 90%	18.2	-7.4 (-9.0 to -5.7)	5.5 (2.8 to 8.3)
ICU occupancy[d]	57.4	-3.1 (-4.0 to -2.2)	38.9 (34.7 to 43.2)
ICU occupancy[d] ≥ 75%	19.6	-2.0 (-3.9 to -0.2)	49.2 (45.7 to 52.7)
ICU occupancy[d] ≥ 90%	0.7	2.1 (1.0 to 3.1)	44.5 (40.5 to 48.4)

[a] Data are from the Agency for Healthcare Research and Quality Healthcare Cost and Utilization Project State Inpatient Databases (2019-2020).
[b] Regressions compared outcomes for each hospital-week of 2020 to the corresponding hospital-week in 2019, i.e., regression models included hospital-week fixed effects. Means and regressions were weighted by the number of admissions for each hospital in 2019.
[c] Inpatient occupancy measured per 100 beds and estimated using data from 45 states.
[d] ICU occupancy measured per 100 beds and estimated using data from 33 states.
[e] Low COVID-19 admission rate corresponds to less than 1 weekly COVID-19 admission per 100 beds.
[f] High COVID-19 admission rate corresponds to 15 or more weekly COVID-19 admissions per 100 beds.

eTable 8. Changes in Occupancy by Service Line During 2020, Full Regression Results[a,b]

	Maternal	M/SUD[d]	Injury	Surgical	Medical
	Percentage-point change in occupancy per 100 beds (95% CI)				
Weeks 1-10* COVID[c] <1	0.0 (-0.1 to 0.1)	0.0 (-0.0 to 0.1)	0.1 (0.0 to 0.1)	-0.2 (-0.3 to -0.1)	0.7 (0.5 to 0.9)
Weeks 1-10* COVID[c] ≥1	-0.2 (-0.7 to 0.3)	0.2 (-0.1 to 0.5)	-0.1 (-0.2 to 0.1)	-0.6 (-1.1 to -0.0)	1.0 (0.1 to 1.9)
Weeks 11-26* COVID[c] <1	-0.6 (-0.7 to -0.5)	-0.7 (-0.8 to -0.6)	-0.4 (-0.4 to -0.3)	-3.7 (-3.9 to -3.5)	-7.5 (-7.7 to -7.2)
Weeks 11-26* COVID[c] 1-4	-0.9 (-1.0 to -0.7)	-0.7 (-0.8 to -0.6)	-0.5 (-0.5 to -0.4)	-4.6 (-4.8 to -4.4)	-6.8 (-7.0 to -6.5)
Weeks 11-26* COVID[c] 5-10	-0.9 (-1.0 to -0.7)	-0.9 (-1.1 to -0.8)	-0.6 (-0.7 to -0.5)	-5.1 (-5.4 to -4.8)	-3.9 (-4.3 to -3.5)
Weeks 11-26* COVID[c] 10-15	-0.9 (-1.1 to -0.7)	-1.3 (-1.5 to -1.0)	-0.8 (-0.9 to -0.7)	-6.0 (-6.6 to -5.4)	-0.2 (-0.9 to 0.5)
Weeks 11-26* COVID[c] 15+	-1.1 (-1.3 to -0.8)	-2.3 (-2.8 to -1.9)	-1.2 (-1.3 to -1.0)	-8.5 (-9.3 to -7.6)	10.7 (9.5 to 11.9)
Weeks 27-48* COVID[c] <1	-0.9 (-1.1 to -0.7)	-0.4 (-0.7 to -0.2)	0.0 (-0.1 to 0.1)	-1.2 (-1.4 to -1.0)	-3.3 (-3.7 to -2.9)
Weeks 27-48* COVID[c] 1-4	-0.9 (-1.1 to -0.8)	-0.3 (-0.4 to -0.2)	0.1 (0.0 to 0.2)	-0.8 (-1.0 to -0.7)	-1.2 (-1.5 to -0.9)
Weeks 27-48* COVID[c] 5-10	-0.9 (-1.0 to -0.8)	-0.4 (-0.5 to -0.3)	0.1 (0.0 to 0.1)	-1.0 (-1.2 to -0.9)	2.2 (1.9 to 2.4)
Weeks 27-48* COVID[c] 10-15	-0.7 (-0.9 to -0.6)	-0.4 (-0.5 to -0.3)	-0.1 (-0.1 to -0.0)	-1.5 (-1.7 to -1.3)	5.7 (5.3 to 6.0)
Weeks 27-48* COVID[c] 15+	-0.7 (-0.9 to -0.5)	-0.3 (-0.4 to -0.2)	-0.2 (-0.3 to -0.1)	-2.3 (-2.5 to -2.0)	12.4 (11.9 to 13.0)
Mean of 2019 occupancy	11.1	5.7	4.2	19.7	39.4
N	374880	374880	374880	374880	374880

© 2023 Meille G et al. *JAMA Health Forum*.

[a] Data are from the Agency for Healthcare Research and Quality Healthcare Cost and Utilization Project State Inpatient Databases (2019-2020) for 45 US states.
[b] Regressions compared outcomes for each hospital-week of 2020 to the corresponding hospital-week in 2019, i.e., regression models included hospital-week fixed effects. Means and regressions were weighted by the number of admissions for each hospital in 2019.
[c] COVID denotes weekly COVID-19 admissions per 100 beds.
[d] M/SUD denotes mental health and substance use disorders.

© 2023 Meille G et al. *JAMA Health Forum*.

eFigure 1. Distribution of Duration of Periods With High COVID-19 Admissions[a,b]

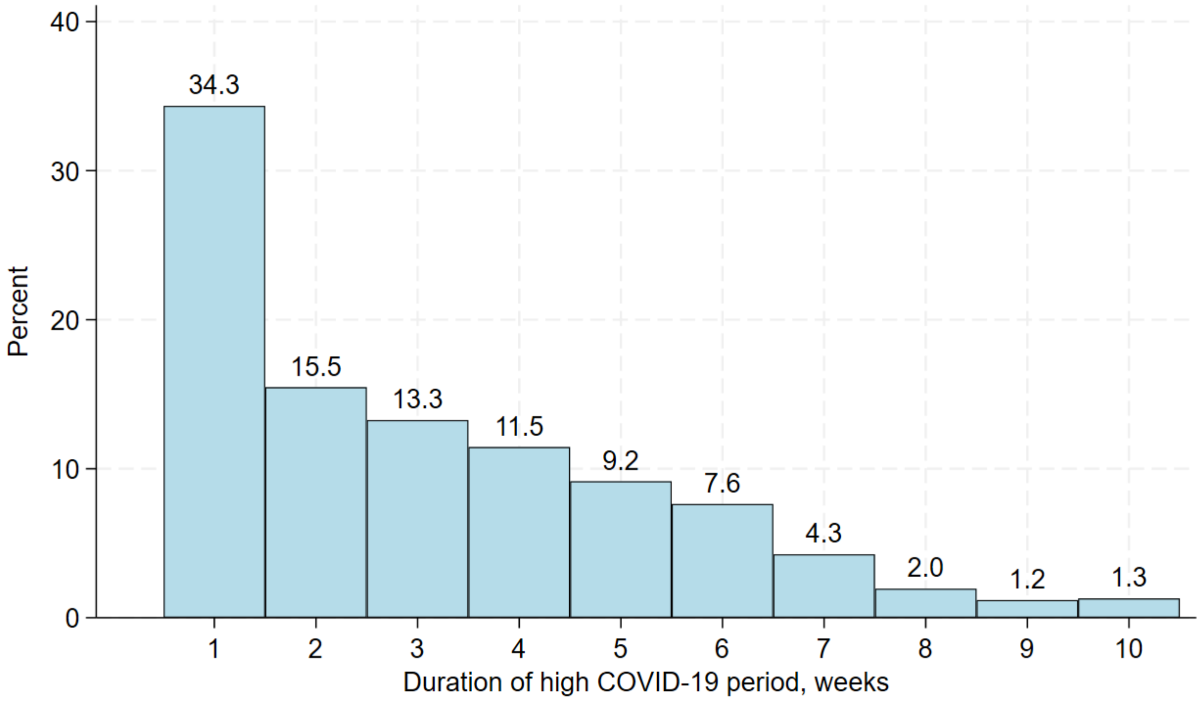

[a] Data are from the Agency for Healthcare Research and Quality Healthcare Cost and Utilization Project State Inpatient Databases (2019-2020) for 45 US states.

[b] A period of high COVID-19 is defined as a set of consecutive weeks with high COVID-19 admissions for a given hospital. Duration is calculated starting from the first week with high COVID-19 in each period. Each hospital may contribute more than one period with high COVID-19. Weighted by number of admissions in 2019 for each hospital. Time frame: 2020, weeks 1-48. More than 10 weeks mapped to 10.

© 2023 Meille G et al. *JAMA Health Forum*.

eFigure 2. Percentage of Hospitals With High COVID-19 Admission Rates by Week in 2020[a,b,c]

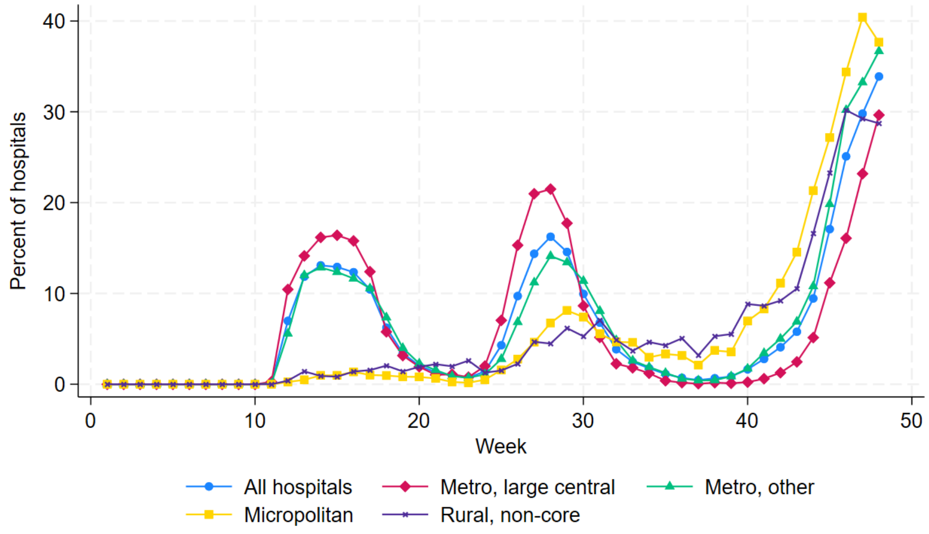

[a] Data are from the Agency for Healthcare Research and Quality Healthcare Cost and Utilization Project State Inpatient Databases (2019-2020) for 45 US states.
[b] High COVID-19 admission rate corresponds to 15 or more weekly COVID-19 admissions per 100 beds.
[c] Weighted by number of admissions in 2019 for each hospital.

© 2023 Meille G et al. *JAMA Health Forum*.
