# Supplementary material for: COVID-19 Admission Rates and Changes in US Hospital Inpatient and Intensive Care Unit Occupancy
Source: JAMA Health Forum. 2023 Dec 1;4(12):e234206. doi: 10.1001/jamahealthforum.2023.4206 (PMC10692846; doi:10.1001/jamahealthforum.2023.4206)
Supplement: Supplement 2. — Data Sharing Statement [file jamahealthforum-e234206-s002.pdf]

Data Sharing Statement

Meille. COVID-19 Admission Rates and Changes in US Hospital Inpatient and Intensive Care Unit Occupancy. *JAMA Health Forum*. Published December 01, 2023. doi:10.1001/jamahealthforum.2023.4206

Data
Data available: No

Additional Information
Explanation for why data not available: This study used restricted data from the Healthcare Cost and Utilization Project (HCUP). It contained exact admission dates, which cannot be publicly shared because of restrictions in the data use agreement between the Agency for Healthcare Research and Quality and HCUP partner states.
